# Use of Antibiotic Lavage in Total Knee Replacement to Prevent Postoperative Infection

**DOI:** 10.7759/cureus.32727

**Published:** 2022-12-20

**Authors:** Abdulaziz Almaawi, Ghazi Aldalbahi, Sara N Albqami, Abdulrahman Barri, Mada Albatly, Orfan Arafah

**Affiliations:** 1 Orthopaedic Surgery, College of Medicine, King Saud University, Riyadh, SAU; 2 Orthopaedic Surgery, King Saud Medical City, Riyadh, SAU; 3 Internal Medicine Department, College of Medicine, King Saud University, Riyadh, SAU; 4 Emergency Department, College of Medicine, King Saud University, Riyadh, SAU

**Keywords:** tkr, vancomycin lavage, periprosthetic joint infection, septic arthritis, surgical wound infection, arthroplasty

## Abstract

Purpose

To determine the effectiveness of using antibiotic lavage in preventing postoperative infections in total knee replacement (TKR) patients.

Methods

Data on all patients who underwent TKR, either primary or secondary, during the period from May 2015 to April 2019 were collected. Many factors (both patient-related and surgery-related) that can increase the risk of surgical site infection (SSI) were taken into consideration to eliminate confounding factors.

Results

A total of 685 patients were identified; out of those, 232 patients received intraoperative antibiotic lavage and 453 did not. We noted that out of all 13 patients who developed SSI, only one patient (7.7%) had received antibiotic lavage, while the other 12 (92.3%) patients did not receive antibiotic lavage. However, the difference was not statistically significant (p=0.078).

Conclusion

Using intraoperative vancomycin lavage was associated with a decrease in the incidence of SSI post-TKR, but the difference was not statistically significant. However, more studies are needed in this area.

## Introduction

Lower limb arthroplasty is a common surgical procedure performed on a daily basis, especially total knee replacement (TKR). In 2030, the estimated number of TKR cases in the USA will be 3.48 million [1]. The implantation of a large foreign body increases the risk of a deep surgical site infection (SSI) and emerges the need for effective perioperative strategies. Prosthetic joint infections (PJI) are one of the most feared complications in orthopedic surgery. Even with antiseptic techniques, SSI and PJI are common complications after orthopedic surgery, which might lead to unpleasant and unfavorable consequences such as a higher rate of prosthesis failure, hospitalization, and morbidity [2,3]. There are a multitude of risk factors that are attributed to the increased chance of the development of SSIs and PJIs. Most risk factors are attributed to host factors like the presence of underlying diseases such as obesity, diabetes mellitus (DM), rheumatoid arthritis, preoperative anemia, and cardiovascular diseases [4-11]. Surgical factors can also increase the risk of infection with longer operative duration and hospital stay [12-17]. Also, Minnema et al. and Wymenga et al. suggested that a high American Society of Anesthesiologists score contributes to the development of SSIs [18,19].

Infection is a devastating complication that bothers both orthopedic surgeons and patients. It can have a big impact on health care costs and length of stay; that is why many surgeons consider intraoperative antibiotic use as an important step in reducing the infection rate in TKR; however, it still remains controversial internationally [11]. The prevention of PJIs and SSIs mostly depends on controlling patients’ underlying diseases and optimizing their condition. In a study conducted on diabetic patients, it was found that regardless of the diabetes type, if the blood sugar was uncontrolled, it doubled the infection rate and led to a significant increase in surgical complications and an increase in the mortality rate [9]. Despite guideline recommendations, several studies explored the use of intraoperative antibiotics such as vancomycin, which has shown the ability to reduce the infection rate by up to 50% and was found to be highly effective in the reduction of SSIs [20,21]. However, Brown et al. showed that the use of antibiotics has no benefit over normal saline in wound irrigation [22]. For an antibiotic agent to be used during irrigation, it should possess a broad spectrum of antimicrobial activity, be used frequently, be used with pulsatile lavage systems, and be left in the wound for approximately one minute before removal [23]. In this study, we attempted to determine whether the use of antibiotic lavage in TKR can reduce postoperative infection.

## Materials and methods

Setting and study population

Approval from the institutional review board at King Saud University - College of Medicine was obtained prior to starting the study. The study was also conducted according to the principles of the Helsinki Declaration. This is a retrospective observational study conducted from May 2015 to April 2019 at King Saud University Medical City (KSUMC) in Riyadh, Saudi Arabia. We included all patients who underwent either primary or secondary TKR during the study period. All data were extracted from the KSUMC electronic database using surgical terminology codes, and patients were then subdivided into two groups: patients receiving intraoperative antibiotic lavage and patients not receiving antibiotic lavage intraoperatively.

All patients received standard antibiotic prophylaxis with 2 g of cefazolin IV 30 minutes preoperatively. The antibiotic group received a lavage consisting of 2 g of vancomycin powder diluted in 2 L of normal saline (1000 mg/1 L) and irrigation was done after the installation of the prosthesis during the cementation period for around 15 minutes prior to the closure of the capsule. The dose of vancomycin is based on the current literature, which shows a safe and minimal toxic effect of vancomycin on osteoblast replication at the cellular level with a concentration of 1000 mcg/ml or less [24]. The no-antibiotic group received 2 L of normal saline irrigation alone after the installation of the prosthesis and prior to the closure of the capsule. In the postoperative period, all patients from both groups received a standard dose of 1 g of cefazolin for three doses. Patients were followed up in the clinic from two weeks to six months after the surgery. Surgical wounds were checked in every visit, and clips and sutures were removed two weeks postoperatively.

Data collection 

Many factors (both patient-related and surgery-related) can increase the risk of SSI; thus, we collected further information on demographics, the patient’s comorbidities, surgical procedural duration, antibiotic prophylaxis use pre and postoperatively, type of procedure (unilateral or bilateral TKR), and whether the patient had an intraoperative or postoperative complication and if they developed an infection.

Data analysis

The analysis was performed using Statistical Package for Social Sciences version 22.0 software (SPSS, Inc., Chicago, IL, US) to determine the demographics and the value of using vancomycin lavage intraoperatively. Categorical data were expressed using frequency and percentage. A chi-square and Fischer’s exact test were used to compare the categorical data for the two groups: those who took an antibiotic and those who did not. We assumed statistical significance when the p-value was less than 0.05.

## Results

The sample size of our study was 685 and included all patients who underwent primary TKR during our set study period from May 2015 to April 2019. Those patients were divided into two groups. The groups were determined by whether they received antibiotic lavage intraoperatively or not. The number of patients who did not receive antibiotic lavage intraoperatively was 453 (66.1%), and the number of patients who received vancomycin lavage intraoperatively was 232 (33.8%). Most of our patients were female: 351 of those who did not receive antibiotics were female (77.5%), and 117 of those who did receive them were also female (50.4%) (Table 1).

**Table 1:** Summary of demographic data and risk factors BMI: body mass index, DM: diabetes mellitus, HTN: hypertension, RA: rheumatoid arthritis.

Variable	Value	No antibiotic lavage given (n = 453)	Antibiotic lavage given (n = 232)	P-value	OR (95% CI)
Gender	Male	102 (22.5%)	115 (49.6%)	0.416	1.21 (0.76, 1.94)
	Female	351 (77.48%)	117 (50.4%)		
Age	≤50 years	22 (4.85%)	13 (5.6%)	0.675	0.86 (0.42, 1.74)
	>50 years	431 (95.14%)	219 (94.39%)		
Duration of surgery	≤120 min	99 (21.85%)	27 (11.63%)	0.001	2.12 (1.34, 3.36)
	>120 min	354 (78.14%)	205 (88.36%)		
DM		209 (46.13%)	92 (39.65%)	<0.0001	1.97 (1.41, 2.76)
HTN		255 (56.29%)	129 (55.6 %)	0.020	1.48 (1.06, 2.07)
Hypercholesteremia		170 (37.52%)	85 (36.63 %)	0.017	1.51 (1.08, 2.11)
Smoking		5 (1.1%)	5 (2.155%)	0.662	0.76 (0.22, 2.64)
Cancer		14 (3.09%)	11 (4.74 %)	0.908	0.95 (0.42, 2.14)
RA		21 (4.63%)	18 (7.75 %)	0.610	0.84 (0.44, 1.62)
Obesity		363 (80.13%)	194 (83.62 %)	0.289	0.80 (0.52, 1.21)
Infectious diseases		23 (5.07%)	9 (3.87 %)	0.099	1.94 (0.88, 4.29)
Enteropathies		68 (15%)	42 (18.10 %)	0.451	1.18 (0.77, 1.81)

When further evaluating the efficacy of the antibiotic lavage among those who underwent TKR, we noted that using the antibiotics lavage in those who underwent TKR replacement did not significantly reduce infections postoperatively. Out of our entire sample size of 685, only 13 had infections. Twelve of those patients were among the group who did not receive antibiotics (6.18%); the remaining one was from the other group (0.43%) (p=0.078) (Table 2).

**Table 2:** Summary of operative data

Variables		No antibiotic lavage given (n = 453)	Antibiotic lavage given (n = 232)	P-value	OR (95% CI)
Location	Unilateral	364 (80.35%)	159 (68.53%)	0.308	1.27 (0.80,1.99)
	Bilateral	87 (19.2%)	30 (12.93%)		
Infection		12 (6.18%)	1 (0.43%)	0.078	6.29 (0.81,48.64)
Intraoperative complications		10 (2.2%)	10 (4.3%)	0.131	0.50 (0.21,1.23)
Postoperative complications		40 (8.83%)	31 (13.36%)	0.064	0.62 (0.38,1.03)

Of all patients, 650 (94.9%) were above the age of 50, which constitutes the majority of patients in both groups, 431 (95.14%) and 219 (94.39%), respectively (p=0.675).

## Discussion

This is the first study, to the best of our knowledge, in Saudi Arabia to report the effect of the use of antibiotic lavage in TKR to prevent postoperative infection. Our study found that the infection rate was 1.89% in all patients (those with and without antibiotics) and showed a non-significant decrease in the incidence of SSI post-TKR (P=0.078). A similar study done by Conroy et al. showed no benefit from the use of antibiotic solutions over normal saline [25]. On the other hand, after the administration of diluted betadine lavage, the rate of infection was reduced from 0.97% to 0.15%, with a significant difference in the rate of infection (p = 0.04). Diluted betadine 10% solutions were found to decrease postoperative infectious complications in orthopedic procedures, including postoperative infections in TKR, in other studies and in other types of surgeries, with minimal side effects [26-28]. Betadine use was found to be a safe and inexpensive choice that is present in almost all operating rooms [27] and can be cost-effective in preventing infections in TKR [29].

Other studies have achieved a drop in the rate of infection by using a high concentration of antibiotics at the time of implant insertion, even with prolonged operation times or in revision procedures [21,29]. A meta-analysis that included 15 studies in different surgical specialties showed that the use of prophylactic antibiotic lavage was found to be effective over the use of saline, water, or no irrigation in 10 studies, while the other five showed no difference [30].

According to our findings, there was no association between the comorbidities or patient-related factors and the increase in infection rate except for DM, which was controlled by multivariate analysis. This is similar to a study conducted on diabetic patients, which found that if the blood sugar was uncontrolled, it doubled the infection rate in patients after total joint arthroplasty [9].

Limitations of the study

There are a couple of weaknesses in our study. First, this is a retrospective cohort study, which is subjected to inconsistency when it comes to the process of data collection. However, the prospective nature of our data collection may have played a role in reducing recall and selection bias. Second, the small sample size of our study, which included only 685 patients.

## Conclusions

Using intraoperative vancomycin lavage was associated with a decreased incidence of SSI post-TKR, but there was no statistically significant difference. The multivariate analysis, after adjusting other variables in the model, showed that only DM was independently related to the outcome. For the establishment of a gold standard in intraoperative prophylactic antibiotic lavage, we recommend that a prospective randomized control trial aiming to prevent postoperative infections be conducted. That would eliminate all questions and show the difference in costs and antibiotic use's adverse effects.
